# Febrile Seizures Cause a Rapid Depletion of Calcium-Permeable AMPA Receptors at the Synapses of Principal Neurons in the Entorhinal Cortex and Hippocampus of the Rat

**DOI:** 10.3390/ijms241612621

**Published:** 2023-08-09

**Authors:** Tatyana Y. Postnikova, Alexandra V. Griflyuk, Arseniy S. Zhigulin, Elena B. Soboleva, Oleg I. Barygin, Dmitry V. Amakhin, Aleksey V. Zaitsev

**Affiliations:** Sechenov Institute of Evolutionary Physiology and Biochemistry of RAS, 44, Toreza Prospekt, Saint Petersburg 194223, Russia; tapost2@mail.ru (T.Y.P.); griflyuk.al@mail.ru (A.V.G.); arseniy.zhigulin@yandex.ru (A.S.Z.); soboleva.elena.1707@gmail.com (E.B.S.); oleg_barygin@mail.ru (O.I.B.); dmitry.amakhin@gmail.com (D.V.A.)

**Keywords:** febrile seizures, hyperthermia, hippocampus, GluA2-lacking AMPA receptor, entorhinal cortex, IEM-1460, development

## Abstract

Febrile seizures (FSs) are a relatively common early-life condition that can cause CNS developmental disorders, but the specific mechanisms of action of FS are poorly understood. In this work, we used hyperthermia-induced FS in 10-day-old rats. We demonstrated that the efficiency of glutamatergic synaptic transmission decreased rapidly after FS by recording local field potentials. This effect was transient, and after two days there were no differences between control and post-FS groups. During early ontogeny, the proportion of calcium-permeable (CP)-AMPA receptors in the synapses of the principal cortical and hippocampal neurons is high. Therefore, rapid internalization of CP-AMPA receptors may be one of the mechanisms underlying this phenomenon. Using the whole-cell patch-clamp method and the selective CP-AMPA receptor blocker IEM-1460, we tested whether the proportion of CP-AMPA receptors changed. We have demonstrated that FS rapidly reduces synaptic CP-AMPA receptors in both the hippocampus and the entorhinal cortex. This process was accompanied by a sharp decrease in the calcium permeability of the membrane of principal neurons, which we revealed in experiments with kainate-induced cobalt uptake. Our experiments show that FSs cause rapid changes in the function of the glutamatergic system, which may have compensatory effects that prevent excessive excitotoxicity and neuronal death.

## 1. Introduction

Febrile seizures (FSs) are one of the most common neurological disorders in childhood [1,2,3]. Short febrile seizures (<10 min) are considered relatively harmless, but complex seizures lasting more than 15 min can lead to several neurological disorders in the future [4]. These include the development of pharmacoresistant temporal lobe epilepsy [2,5,6].

Data from an experimental animal model of FS have also shown that prolonged FS leads to long-term changes in the properties of hippocampal neurons with critical consequences for the excitability of the hippocampal network [7]. These changes further increase the susceptibility of animals to limbic seizures throughout life [8]. Evidence from other neonatal seizure models (hypoxia- or flurotil-induced seizures) strongly supports these findings and shows that seizures early in life cause irreversible functional changes in neural networks, rendering the brain vulnerable to later epilepsy and leading to cognitive impairment [9,10,11]. Even a single neonatal seizure can significantly alter the properties of the glutamatergic synapses [12]. However, the specific mechanisms that mediate changes in synaptic properties following seizures remain largely unknown.

One of the common consequences of epileptic seizures is alterations in the number and subunit composition of ionotropic glutamate AMPA receptors, which have been found both in the brains of epileptic patients and in a number of experimental animal models [13,14,15,16,17]. Glutamate AMPA receptors consist of four homologous pore-forming subunits (GluA1–GluA4) that are predominantly assembled into diheteromers [18]. The functional properties of AMPA receptors depend on the presence of the GluA2 subunit, the absence of which or the presence of an unedited GluA2 subunit renders the receptor permeable to Ca^2+^ ions [19,20,21].

Many studies have shown that seizures result in changes in the proportion of CP-AMPA receptors. Most of the investigations have demonstrated an increase in CP-AMPA receptor expression [22,23,24,25,26,27]. The insertion of CP-AMPA receptors during seizures may result in an increase in intracellular calcium concentration with excessive activation of glutamatergic inputs, and may even be a cause of neuronal death [28]. Disruptions in the number and composition of AMPA receptors are thought to underlie subsequent cognitive deficits [21,29]. However, there are studies suggesting a decrease in the expression of CP-AMPA receptors [30,31]. Thus, changes in the subunit composition of AMPA receptors and the appearance of CP-AMPA receptors depend on many factors (age of the animal, type of model, time after seizures, etc.). Age may be a key factor in altering the subunit composition of glutamate receptors. In the early stages of development, CP-AMRA receptors are widely expressed in the CNS [20]. But as the animal matures, the expression of CP-AMRA receptors in synapses decreases significantly, and in the brain of adult animals, CP-AMRA receptors are mainly found in interneurons and glial cells [20,21,32,33].

FSs are observed at an age when the expression of CP-AMPA receptors in pyramidal neurons is high, and their hyperactivation during seizures may cause excitotoxicity. However, FSs usually do not cause significant neuronal death, which has been shown in several studies [34,35,36]. Therefore, the aim of our work was to investigate the properties of glutamatergic transmission and to determine the proportion of CP-AMPA receptors in the synapses of the rat hippocampus and entorhinal cortex during the acute phase (in brain slices prepared 15 min after FSs) and during the delayed phase (48 h) after FSs. FSs lasting at least 15 min were induced by hyperthermia in 10-day-old (P10) rats.

## 2. Results

### 2.1. The Efficacy of Synaptic Neurotransmission at CA3–CA1 Is Immediately Reduced after FSs

**Figure 1:**
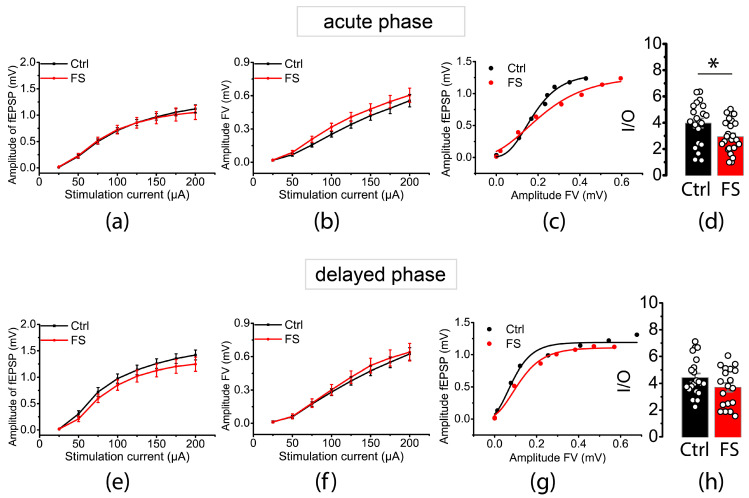
Stimulus–response relationships for fEPSP amplitudes (**a**,**d**) and presynaptic fiber volley (FV) amplitudes (**e**,**f**) recorded from hippocampal CA1 area in slices prepared 15 min ((**a**–**d**), acute phase) and 48 h ((**e**–**h**), delayed phase) after FSs. According to repeated measures ANOVA, the magnitude of fEPSP amplitude (**a**,**e**) in rat hippocampal slices did not differ from that in control animals during either the acute (F_7,294_ = 0.22, *p* = 0.98) or delayed phase (F_7,266_ = 0.96, *p* = 0.46) after FSs. FV amplitudes did not change significantly either during the acute (F_7,294_ = 0.43; *p* = 0.88) or delayed phase (F_7,266_ = 0.14, *p* = 0.99) after FSs. Each point represents the mean ± standard error of the mean. (**c**,**g**) Representative examples of I/O relationships between the fEPSP and FV amplitudes in hippocampal slices. The maximum I/O slope is significantly reduced during the acute phase (**d**), but not during delayed phase (**h**) after FS; *t*-test: *—*p* < 0.05. Ctrl—control rat, FS—post-FS rats.

We first investigated whether FSs affect glutamatergic synaptic neurotransmission in CA3–CA1 of the rat hippocampus. FSs were induced in P10 rats. Hippocampal slices were prepared at two time points: 15 min (*n* = 23 slices, *N* = 9 rats) and 48 h after FSs (*n* = 19, *N* = 9). We compared the results with age-matched control animals (P10: *n* = 21, *N* = 10; P12: *n* = 21, *N* = 10). We measured the amplitudes of fEPSPs and presynaptic fiber volleys (FVs) recorded at the *stratum radiatum* of CA1 as a function of extrasynaptic stimulation intensity (25–200 μA, Figure 1a,b,e,f). We then plotted the amplitudes of fEPSPs (mainly reflecting the activation of postsynaptic AMPA receptors) against the amplitudes of FV (mainly reflecting the number of CA3 axons that evoked the action potential) for each slice (Figure 1c,g).

The composite cellular transfer function between the action potentials of the presynaptic neuron and the response of the postsynaptic membrane to glutamate release is reflected by the slopes of these input–output (I/O) relationships. Therefore, the maximum I/O slopes can be used to measure basal synaptic transmission. By fitting the curves with the sigmoidal Gompertz function, we calculated the maximum I/O slopes and found that the I/O curve slopes were significantly smaller in post-FS animals than control animals during the acute phase (control: 4.0 ± 0.4, *n* = 21; control: 2.9 ± 0.3, *n* = 23; *t* = 2.4; *p* < 0.05; Figure 1d) but not during the delayed phase after FSs (control: 4.4 ± 0.3, *n* = 21; control: 3.7 ± 0.3, *n* = 19; *t* = 1.38; *p* = 0.18; Figure 1h).

The results indicate that FSs lead to a swift reduction in the synaptic transmission efficacy in the hippocampus. This may happen as a compensatory reaction to the overstimulation of glutamatergic transmission during FSs.

### 2.2. FSs Cause a Rapid Decrease in the Proportion of CP-AMPA Receptors in the Principal Neurons of the Entorhinal Cortex and Hippocampus

The entorhinal cortex is a major output of the hippocampal CA area and interactions between the entorhinal cortex and hippocampus play a significant role in the generation of epileptic activity [37,38]. Since the proportion of CP-AMPA receptors in the synapses of principal cortical and hippocampal neurons is high during early ontogeny, we tested whether the proportion of CP-AMPA receptors changes. To assess this, we first examined the properties of evoked EPSCs in deep entorhinal pyramidal neurons under local electrical stimulation. For this purpose, AMPA-mediated eEPSCs were isolated pharmacologically with NMDA and GABAa receptor antagonists (50 μM AP5, 10 μM MK-801, 10 μM gabazine). The stimulation current was chosen to elicit a monosynaptic response with an amplitude of 50–150 pA.

**Figure 2:**
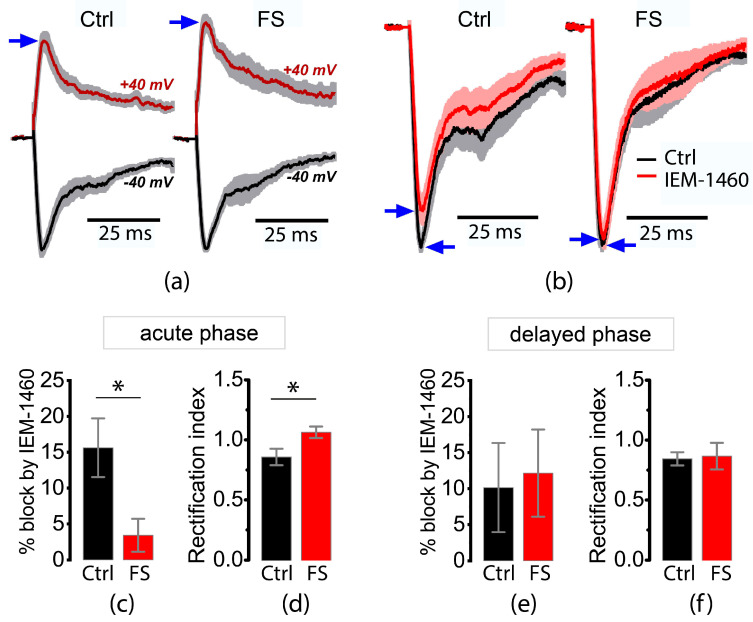
Rapid decrease in the proportion of CP-AMPA receptors in the postsynaptic membrane of entorhinal cortex pyramidal neurons after FSs. (**a**) Normalized and averaged eEPSCs mediated by AMPA receptors were recorded at −40 and +40 mV in rats at P10. The shaded area shows the standard error. The eEPSCs were normalized to the peak amplitude of the negative current. In the control recordings, the negative current had a higher absolute amplitude than the positive, which was not observed in the recordings from the post-FS rats. Blue arrows indicate positive current peaks. (**b**) Normalized, averaged eEPSCs mediated by AMPA receptors were recorded to illustrate the blocking effect of IEM-1460 in P10 rats. The amplitude of eEPSCs was normalized to the peak current recorded prior to the application of IEM-1460. The effect of the antagonist was reduced in rats after FSs. Blue arrows indicate the location of the current peaks. (**c**) The block of eEPSCs by IEM-1460 and (**d**) the rectification index of eEPSCs in P10 rats. IEM-1460 exerts a weaker block of eEPSCs in the post-FS group compared to age-matched controls (*t*-test, * *p* = 0.01, *n* = 14 and 16 for control and post-FS groups, respectively). The rectification index was greater in post-FS rats compared to controls (*t*-test, * *p* = 0.02, *n* = 15 and 19 for control and post-FS groups, respectively); (**e**) block of eEPSCs by IEM-1460 and (**f**) rectification index of eEPSCs in P12 rats. No significant differences were found between control and post-FS groups (*n* = 5 and 5 for (**e**); *n* = 12 and 13 for (**f**)).

Since CP-AMPA receptors exhibit an inwardly rectifying current–voltage relationship, we performed whole-cell patch-clamp recordings of eEPSCs at −40 mV and +40 mV and calculated the rectification index as the ratio of eEPSC amplitudes at +40 and −40 mV (Figure 2a). Next, we evaluated the blocking effect of IEM-1460, the selective antagonist of CP-AMPA receptors (Figure 2b). In the acute phase after FS, the rectification index of the eEPSC was significantly higher in post-FS rats than in control rats (Figure 2d,f), and the application of IEM-1460 induced a significantly greater block of AMPA-mediated eEPSCs in control rats than in rats subjected to FS (Figure 2c,e).

However, no significant change in eEPSC block and rectification index was observed during the delayed phase after FSs (Figure 2e,f). These results show that FSs quickly reduce the presence of CP-AMPA receptors in the synapses of the principal neurons of young rats.

Since only synaptic AMPA receptors were activated in this experiment, we can assume that CP-AMPA receptors were translocated from the postsynaptic density to the extrasynaptic region of the membrane or were internalized within neurons. To determine which of these possibilities is more likely, we performed an additional study in isolated neurons. In this experiment, we used isolated pyramidal neurons from the CA1 region of the hippocampus and a rapid extracellular application of kainate, which allowed us to activate all AMPA receptors on the cell membrane.

**Figure 3:**
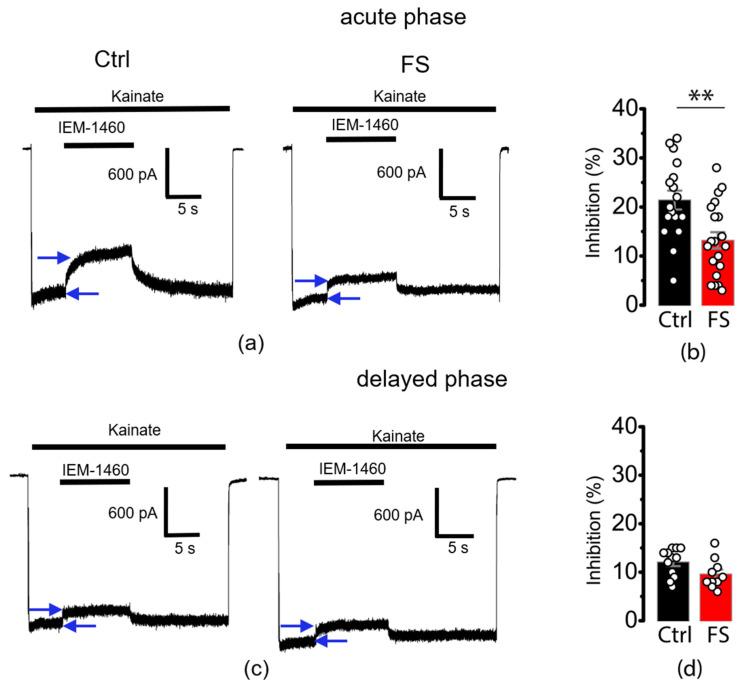
Inhibition of kainate-induced currents in isolated pyramidal neurons from CA1 hippocampus by IEM-1460. Representative examples of inhibition of CP-AMPA receptors (**a**) during acute and (**c**) delayed phase after FSs. Blue arrows indicate the current that is mediated by CP-AMPA receptors. Mean percentage blockade by IEM-1460 (100 μM) of kainate-induced currents (**b**) during acute and (**d**) delayed phase after FSs. Each point represents one value obtained for each neuron. All data are presented as mean ± standard error of the mean; *t*-test: ** *p* < 0.01.

We showed that the application of 100 µM kainate to isolated hippocampal CA1 pyramidal neurons from control and post-FS rats induced similar weakly desensitizing inward currents. We compared the percentage of inhibition of kainate-induced currents by the selective CP-AMPA receptor channel blocker IEM-1460 in neurons from control and post-FS rats. Representative examples of kainate-induced current inhibition at −80 mV holding voltage by 100 µM IEM-1460 are shown in Figure 3a,c.

In experiments with neurons from P10 rats, the percentage of inhibition by IEM-1460 was reduced in post-FS animals compared to the control (post-FS: 13.2 ± 1.7%, *n* = 20 vs. control: 21.4 ± 1.9%, *n* = 17, *t* = 3.2, *p* < 0.01). These results indicate that seizures in P10 rats decreased the proportion of CP-AMPA receptors on the membrane surface of hippocampal pyramidal neurons. This supports the notion that seizures induce rapid internalization of CP-AMPA receptors. In contrast, in P12 rats, the percentage of inhibition by IEM-1460 was similar in control and post-FS groups (12.1 ± 0.8%, *n* = 12 vs. 9.6 ± 1.0%, *n* = 10, *t* = 1.97, *p* = 0.06). Thus, in P12 rats, an age-related decrease in the proportion of CP-AMPA receptors in control animals compensated for the difference between the compared groups.

### 2.3. Kainate-Induced Co^2+^ Uptake in Hippocampal CA1 and CA3 Areas and in the Entorhinal Cortex Is Reduced Immediately after FSs

**Figure 4:**
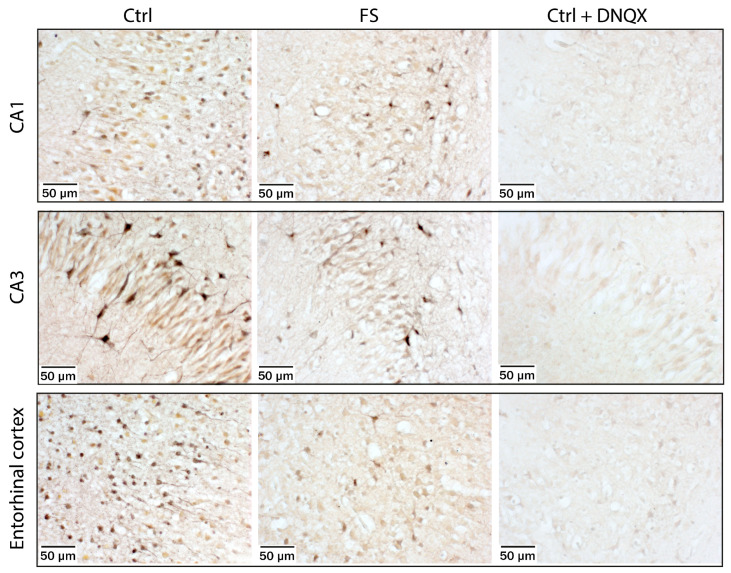
Kainate-induced Co^2+^ uptake showed greater Co^2+^ uptake in hippocampal CA1 and CA3 cell layers and in the entorhinal cortex in acute brain slices from P10 control rats (**left panels**), and poorer Co^2+^ uptake in rats during the acute phase after FSs (**middle panels**). The AMPA and kainate receptor antagonist DNQX (20 μM) completely blocked Co^2+^ uptake in CA1, CA3 and entorhinal cortex of rats (**right panels**).

**Figure 5:**
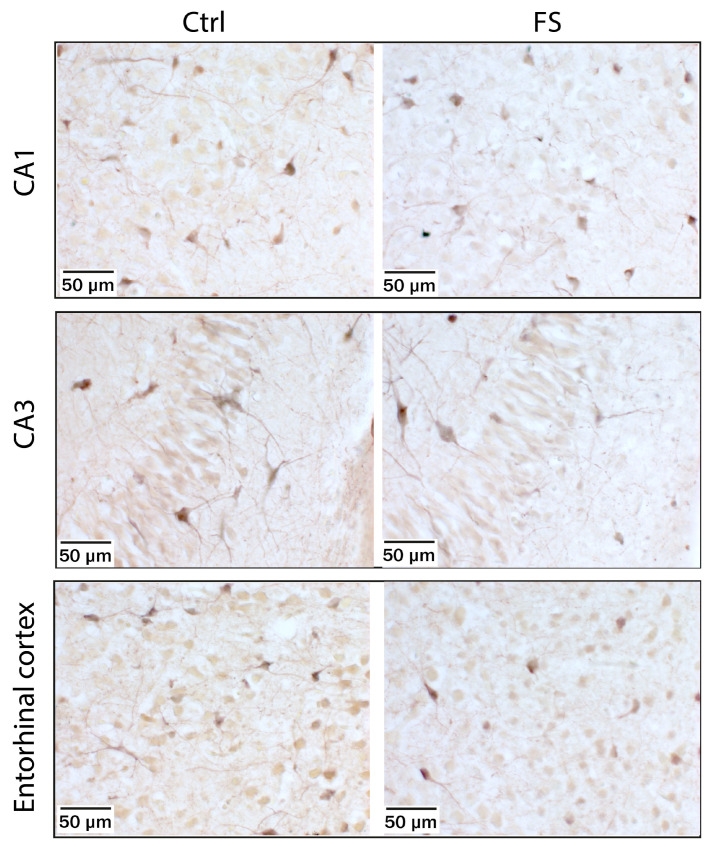
Kainate-induced Co^2+^ uptake showed greater Co^2+^ uptake in hippocampal CA1 and CA3 cell layers and in the entorhinal cortex in acute brain slices from P12 control rats (**left panels**) and poorer Co^2+^ uptake in rats during delayed phase after FSs (**right panels**).

We then used Co^2+^ labeling [25] to directly visualize CP-AMPA receptor-containing cells in rats during the acute (Figure 4) and delayed phase after FSs (Figure 5). Acute brain slices were treated with kainate in the presence of AP-5 and TTX. Co^2+^-stained cells were observed in all areas of the hippocampus and all layers of the entorhinal cortex. In P10 rats, stronger Co^2+^ staining was observed in brain sections from control than from post-FS rats. The morphology of the Co^2+^-stained cells and their location varied. In addition to the cells resembling interneurons known to express CP-AMPA receptors, we saw a significant number of highly stained pyramidal neurons in slices from control animals. In slices from post-FS animals, highly stained pyramidal neurons were virtually absent.

To confirm that the Co^2+^ influx was mediated by AMPA receptors, we performed a control experiment in which the AMPA receptor blocker DNQX was also added to the incubation solution. In this case, we observed a complete absence of Co^2+^-stained neurons (Figure 4). This experiment further confirms that FSs lead to a rapid internalization of CP-AMPA receptors from the membrane of principal neurons.

No differences were observed between P12 control and post-FS rats, either in the CA1 and CA3 areas of the hippocampus or in the entorhinal cortex (Figure 5).

Thus, the histological data confirm the results of the electrophysiological studies and indicate a decrease in CP-AMPA receptors in rats immediately after seizures.

## 3. Discussion

The results obtained indicate that FSs lead to a significant reduction in CP-AMPA receptor presence in the synapses of entorhinal and hippocampal pyramidal neurons of young rats, as shown by whole-cell patch-clamp recordings and measurements of kainate-induced Co^2+^ uptake. These changes affect the functional properties of the neuronal network by reducing baseline synaptic transmission.

Changes in CP-AMPA receptor expression have been reported in several other seizure models. In the lithium–pilocarpine model, an increase in GluA2 expression relative to the expression of other AMPA receptor subunits was found several hours after seizures [30,31]. However, most studies show the opposite. The decrease of GluA2 expression in the hippocampus was found after kainate-induced seizures [22,26]. One week following the pilocarpine-induced seizures, decreased GluA2 gene expression was found in the temporal cortex [39]. The increase of CP-AMPA receptor expression in neocortical principal cells has been observed in in vitro studies [27,40,41].

The present study utilized young P10 rats, which maintain high levels of CP-AMPA receptor expression in the principal neurons of the neocortex and hippocampus [20,33]. It is widely recognized that CP-AMPA receptors are transiently expressed during development in most principal neurons across various brain areas [28,42]. The increased susceptibility of the immature brain to seizures has been hypothesized to be due to the presence of CP-AMPA receptors [33]. Alterations in glutamate receptor subunit composition and expression levels usually take place during the second to third week after rodents are born. The change in expression may occur rapidly. For example, in primary auditory neurons, the switch from GluA2-lacking receptors to GluA2-containing receptors typically occurs around P10 [43]. In other synapses, the predominance of CP-AMPA receptors can shift to calcium-impermeable receptors within a week, as in neocortical layer 5 pyramidal neurons [33], or in hippocampal mossy fiber-CA3 (MF-CA3) pyramidal neuron synapses [44], where the switch occurs within the second to third postnatal weeks.

Therefore, it was unclear whether a FS would further increase CP-AMPA receptor levels or, conversely, cause them to decrease. Using similarly aged rats with high levels of CP-AMPA receptors in principal neurons, it has been shown that hypoxia-induced seizures further increase CP-AMPA receptor expression in the synapses [9,17,25]. The increased expression level of CP-AMPA receptors was observed within days of seizures induced by hypoxia. This was confirmed through both electrophysiological methods (with increased inwardly rectifying current-voltage relationships) and histological methods (with enhanced staining in hippocampal slices for AMPA receptor-mediated Co^2+^ uptake) [25]. The chronic epileptogenic effects of perinatal hypoxia-induced seizures may have contributed to the increased AMPA receptor-mediated Ca^2+^ influx which may also be associated with disturbances in synaptic plasticity that later lead to epilepsy [45,46]. The decrease in the proportion of CP-AMPA receptor which was observed in this study may be unique to the FS model. Compared to the hypoxia-induced seizure model, FSs in the relevant model are less likely to lead to the development of epilepsy later in life. Moreover, the duration of the initial FS was found to influence both the incidence of limbic epilepsy after long FSs and the severity of spontaneous epileptic seizures [8].

Rapid changes in synaptic function are often observed after an epileptic seizure [47]. It can be hypothesized that the transient downregulation of CP-AMPA receptors may be a compensatory mechanism that helps to minimize the consequences of seizures and results in a lack of transition to acquired epilepsy in the model of FS. This hypothesis is supported by a number of data from other studies. For example, both the seizure-induced increase in CP-AMPA receptor function and the concomitant increase in seizure susceptibility in vivo were prevented by the early blockade of AMPA receptors with NBQX [9,17,45] or perampanel [48]. Although the immediate effect of CP-AMPA receptor incorporation into the synapses of the principal neurons of the entorhinal cortex may be antiepileptic [49], the increased expression of CP-AMPA receptors after seizures is usually considered a pathological process that supports the transition from seizures to status epilepticus [50,51].

The fact that “seizure begets seizure” has been confirmed by extensive experimental research [52]. Among the possible mechanisms of epileptogenesis that are related to changes in the level of expression of the CP-AMPA receptors, two main ones can be distinguished. The first is that excessive activity of the CP-AMPA receptors and Ca^2+^ entry through them in neurons that normally express calcium-impermeable AMPA receptors contributes to, or causes, delayed cell death in response to endogenous glutamate [28]. This mechanism is likely to be largely involved in the mature brain, and evidence for this has been obtained in the kainate model of temporal lobe epilepsy and partially in the lithium–pilocarpine model of temporal lobe epilepsy. However, in the immature brain, pyramidal neurons probably have some compensatory mechanisms that prevent their death by excessive activation of CP-AMPA receptors, so that no significant neurodegeneration is observed in either the hypoxic seizure model, which leads to an increase in CP-AMPA receptors, or the febrile seizure model, which leads to a decrease in CP-AMPA receptors. Therefore, we can assume that the alteration of CP-AMPA receptors in early life has no significant effect on neuronal death and is not a key factor in epileptogenesis.

The second mechanism may involve impaired synaptic plasticity. Epileptic seizures are known to induce synaptic plasticity in the hippocampal and neocortical neurons. Several reports indicate that in neurons that do not express CP-AMPA receptors under baseline conditions, seizure-like activity promotes the NMDA receptor-dependent form of synaptic potentiation [27,53,54]. However, our previous results indicate that CP-AMPA receptor activity mostly opposes the long-term enhancement of hippocampal synapses [55], in contrast to its role in conventional LTP formation [56,57]. In rats, hypoxia-induced seizures at P10 cause a rapid increase in miniature EPSC amplitude and frequency in CA1 pyramidal neurons due to increased CP-AMPA receptor expression [9], and a significant decrease in the incidence of silent synapses and persistent attenuation of LTP [58]. Moreover, alterations in CP-AMPA receptor expression may impair long-term depression, which in turn may disrupt normal hippocampal dendritic spine maturation and pruning during development, resulting in an overabundance of less efficient excitatory synapses [17,59].

We can hypothesize that FS in younger rats expressing CP-AMPA receptors may promote a different form of long-term synaptic plasticity compared to that observed in other seizure models. We have previously reported that FSs impair hippocampal LTP due to insufficient NMDAR activity [36]. Several other forms of CP-AMPA receptor-related synaptic plasticity have been described in both principal neurons [60] and interneurons [61]. It was previously reported that high-frequency stimulation of the input to cerebellar stellate cells, which predominantly express CP-AMPA receptors in synapses, leads to the expression of calcium-impermeable AMPA receptors in these cells [62,63,64]. Increased Ca^2+^ entry through synaptic CP-AMPA receptors, but not NMDA receptors, triggers the insertion of GluA2-containing receptors into the synapse and the removal of GluA2-lacking receptors from the synapse. Acute stress can promote the CP-AMPA receptor-dependent form of LTP in the hippocampus [65].

Taken together, the obtained results suggest that the regulatory mechanisms that determine the prevalence of a particular AMPA receptor subtype may be differentially affected by epileptiform activity, depending on the age of the subject and the seizure model. Our study describes a new form of plasticity in synapses of the entorhinal cortex and hippocampus induced by FSs, which is a rapid decrease in the proportion of CP-AMPA receptors. We believe that this form of plasticity may be one of the compensatory mechanisms that provides a decrease in the level of excitation in neuronal circuits and prevents epileptogenesis.

## 4. Materials and Methods

### 4.1. Animals

The experiments were conducted following local guidelines for the treatment of laboratory animals and with obtained approval from the Ethics Committee of the Sechenov Institute of Evolutionary Physiology and Biochemistry. These recommendations fully adhere to the standards for animal research established by Russian and international regulations. The Wistar rat females and pups were kept under standard conditions at room temperature, allowing them access to food and water. Only ten pups were retained in each litter.

### 4.2. FS Model

FSs were induced on postnatal day 10 (P10) as previously described [36,66]. Briefly, the pups were placed at the bottom of a 10 L glass chamber where the temperature was maintained at 46 °C for 30 min. Rectal temperature was measured regularly during the experiments. When the pups’ temperatures rose above 41 °C, they were removed from the chamber and placed on a cool surface for 2 min. In this paradigm, behavioral seizures were stereotyped. They consisted of heat-induced hyperkinetic arrest, followed by facial automatisms, often accompanied by body flexion, and culminating in myoclonic jerks and clonic convulsions.

After inducing hyperthermia, the animals were placed on a cooled surface until their core temperature returned to the normal range. The animals were then returned to their cages. The study involved only animals that had FSs lasting for at least 15 min (N = 30 rats), which corresponded to complex FSs [67]. As controls, littermates were also removed from the cage for the same period, but were kept at room temperature instead (N = 33 rats).

### 4.3. Brain Slice Preparation

The rats were euthanized on the day of testing via decapitation. Subsequently, their brains were immediately and carefully placed in cold (4 °C) oxygenated (95% O_2_/5% CO_2_) artificial cerebrospinal fluid (ACSF). The ACSF contained the following components (in mM): 126 NaCl, 24 NaHCO_3_, 2.5 KCl, 2 CaCl_2_, 1.25 NaH_2_PO_4_, 1 MgSO_4_, and 10 glucose. The preparation of the slices was carried out according to the previously described procedure [68]. A HM 650 V vibratome (Microm International, Walldorf, Germany) was used to prepare horizontal brain slices (350 μm) containing the dorsal hippocampus and the entorhinal cortex (EC), as previously described. The slices were allowed to recover for one hour in oxygenated ACSF at a temperature of 35 °C, prior to the electrophysiological experiments.

### 4.4. Field Potential Recordings

Following incubation, slices were transferred to a recording chamber where they were perfused with a constant flow of artificial cerebrospinal fluid (ACSF) at a rate of 6–7 mL/min at a temperature of 30 °C. We recorded fEPSPs from the CA1 *stratum radiatum* using ACSF-filled glass microelectrodes with a resistance of 0.2–1.0 MΩ. Orthodromic electrical stimulation was used to evoke synaptic responses. A twisted bipolar nichromic stimulation electrode was used in conjunction with an A365 stimulus isolator (World Precision Instruments, Sarasota, FL, USA). The model 1800 amplifier (A-M Systems, Carlsborg, WA, USA) was employed for recording the evoked potentials. Subsequently, the potentials were digitized and stored on a personal computer with the assistance of the NI USB-6211 A/D converter (National Instruments, Austin, TX, USA) and WinWCP v5 software (University of Strathclyde, UK). The electrophysiological data were analyzed using the Clampfit 10.2 software (Axon Instruments, San Jose, CA, USA). We measured the amplitudes of fEPSPs and FVs. Next, we calculated the maximum slope of the input–output (I/O) relationship between fEPSP amplitude and FV amplitude for each slice by fitting a sigmoidal Gompertz function, as previously described [69].

### 4.5. Whole-Cell Patch-Clamp Recordings from Entorhinal Cortex Slices

A Nikon FN-1 microscope (Nikon, Tokyo, Japan) equipped with differential interference contrast optics and a video camera (Grasshopper 3 GS3-U3-23S6M-C; FLIR Integrated Imaging Solutions Inc., Wilsonville, OR, USA) was used to visualize the principal neurons of the deep layers of the medial entorhinal cortex. An aP-1000 pipette puller (Sutter Instrument, Novato, CA, USA) was used to make borosilicate glass pipettes (3–5 MΩ). The pipetting solution contained Cs-methanesulfonate (124 mM), NaCl (10 mM), HEPES (10 mM), EGTA (5 mM), QX314 (6 mM), MK-801 (3 mM), ATP-Mg (4 mM), GTP (0.3 mM), and spermine (0.2 mM), with the pH adjusted to 7.25 by adding CsOH.

Whole-cell patch-clamp recordings were performed using the MultiClamp 700B amplifier (Molecular Devices, Sunnyvale, CA, USA), coupled with the NI USB-6343 A/D converter (National Instruments, Austin, TX, USA), and controlled by WinWCP 5 software (University of Strathclyde, Glasgow, UK). Data were sampled at 40 kHz and low pass filtered at 5 kHz. Typically, the access resistance ranged between 10 and 20 MΩ. For all cells analyzed, the access resistance remained stable throughout the experiment (≤30% increase). We compensated for the liquid junction potential off-line by subtracting 7 mV from the holding voltage value.

We used a bipolar electrode placed between 50 and 150 µm away from the neuron to elicit synaptic responses. Recording of eEPSCs mediated by AMPA receptors was performed in the presence of NMDA and GABAa receptor antagonists (D-AP5, 50 μM; MK-801, 10 μM; gabazine, 10 μM) in the perfusion solution. The rectification index was obtained by calculating the ratio of eEPSC amplitudes recorded at +40 mV and −40 mV. The selective antagonist (IEM-1460, 100 μM) of CP-AMPA receptors was applied to determine their proportion at the synapses of principal neurons.

### 4.6. Patch-Clamp Recordings of Membrane Currents from Isolated CA1 Pyramidal Neurons

Hippocampus sections of 250 µm thickness were prepared using a vibratome (Campden Instruments Ltd., Loughborough, UK) and placed in a solution consisting of the following (in mM): 124 NaCl, 5 KCl, 1.3 CaCl_2_, 2.0 MgSO_4_, 26 NaHCO_3_, 1.24 NaH_2_PO_4_, and 10 D-glucose. The solution was aerated with carbogen. Isolation of single pyramidal neurons from the slices was accomplished through vibrodissociation. The whole-cell patch-clamp technique was employed to record the membrane currents that were produced in response to the application of 100 μM kainate at a holding voltage of −80 mV. A series resistance of approximately 20 MΩ was compensated approximately 70–80% throughout the experiments. The currents were recorded using an EPC-8 amplifier (HEKA Electronics, Lambrecht, Germany). The currents were filtered at 5 kHz, sampled, and saved on a personal computer. The drugs were applied using the RSC-200 perfusion system (BioLogic Science Instruments, Claix, France). The time for solution exchange in whole-cell mode was 50–60 ms. The extracellular solution consisted of (in mM): 143 NaCl, 5 KCl, 2 MgSO_4_, 2.5 CaCl_2_, 18 D-glucose, and 10 HEPES. The pH was adjusted to 7.4 with HCl. The pipette solution contained (in mM): 100 CsF, 40 CsCl, 5 NaCl, 0.5 CaCl_2_, 5 EGTA, and 10 HEPES. The pH was adjusted to 7.2 with CsOH. The experiments were conducted at room temperature of 22–24 °C.

### 4.7. The Kainate-Induced Cobalt Uptake Method

We cut horizontal brain slices (400 μm thick) using an HM 650 V vibratome (Microm, Walldorf, Germany). The sections were allowed to recover in ACSF containing TTX (0.5 μM) for an hour at 28 °C. We conducted all further incubations at a temperature of 20 °C. We transferred the slices to low-sodium, low-calcium Krebs’ solution (consisting of 50 mM NaCl, 2.5 mM KCl, 26 mM NaHCO_3_, 1.25 mM NaH_2_PO_4_, 25 mM glucose, 0.5 mM CaCl_2_, and 2 mM MgCl_2_), containing TTX (0.5 μM) and D-AP5 (100 μM) for 15 min. To serve as a control, we incubated some slices with the AMPA receptor antagonist DNQX (10 μM).

Slices were stimulated with kainate (20 μM) for 20 min in Krebs’ solution containing 1.5 mM CoCl_2_. The slices were then washed for 10 min in a divalent ion-free Krebs’ solution (135 sucrose, 50 NaCl, 2.5 KCl, 26 NaHCO_3_, 1.25 NaH_2_PO_4_, 25 glucose) containing 0.5 mM EDTA. The samples underwent a subsequent rinse of 5 min using the same solution without EDTA. To induce intracellular Co^2+^ precipitation, the slices were incubated with 0.12% (NH_4_)_2_S in divalent ion-free Krebs’ solution for 5 min. After an additional 5 min wash with Krebs’ solution free of divalent ions, the slices were fixed overnight at a temperature of 4 °C in 4% paraformaldehyde (PFA) in 0.1 M phosphate-buffered saline (PBS).

On the following day, the slices were rinsed with 0.1 M PBS. Subsequently, they were incubated in a 30% sucrose solution prepared in 0.1 M PBS. The slices were embedded in Tissue-Tek^®^ O.C.T. Compound (Sakura Finetek USA, Inc., Torrance, CA, USA), frozen in cooled isopentane at a temperature lower than −50 °C (Sigma-Aldrich, St. Louis, MO, USA), and stored at −80 °C for later use. Specimens were sectioned at 20 μm using a Bright OTF5000 cryostat (Bright Instrument Co Ltd., Huntingdon, UK). Sections were placed on microscope slides coated with Super Frost Plus (J1800AMNZ, Fisher Scientific UK Ltd., Loughborough, UK). To enhance the silver, the sections were treated with 2% Na_2_WO_4_ for 10 min. Later, sections were processed in developer solution consisting of 8 parts AgNO_3_ solution (1% Triton X-100, 7.5% CH_3_COOH, 30.3 mM CH_3_COONa, and 2.94 mM AgNO_3_), along with 1 part each of 5% Na_2_WO_4_ and 0.25% ascorbic acid. They were kept in the dark for 15 min and then washed with 2% Na_2_WO_4_. Afterwards, they were dehydrated using increasing concentrations of ethanol solution. The sections were cleared with micro-clearing (Diapath, Martinengo, Italy) and coverslipped with VitroGel (Ergo Production, Moscow, Russia) as the final steps.

Micrographs of stained hippocampal neurons in CA1, CA3, and entorhinal cortex layers from Co^2+^ uptake experiments were obtained using a Zeiss microscope (Carl Zeiss AG, Göttingen, Germany) at ×400 magnification.

### 4.8. Statistical Analysis

We utilized OriginPro 8 (OriginLab Corporation, Northampton, MA, USA) and SigmaPlot 12.5 (Systat Software Inc., Palo Alto, CA, USA) to perform statistical analysis and graphing. We used Dixon’s Q test (with a 90% confidence level) or Iglewicz and Hoaglin’s robust test for multiple outliers (with a modified Z score of at least 3.0) to identify and eliminate any outliers. The Kolmogorov–Smirnov test was used to assess the normality of the sample data. As described in the text, we assessed statistical significance using Student’s *t*-test and ANOVA. We presented all data as mean ± standard error of the mean. We considered *p* < 0.05 as statistically significant.

## Data Availability

The data presented in this study are available on request from the corresponding authors.
